# Effects of a Compound Probiotic on Production Performance, Intestinal Health, Immune Function, and Gut Microbiota in Broiler Chickens

**DOI:** 10.3390/vetsci13030227

**Published:** 2026-02-27

**Authors:** Yuhao Liu, Wenjia Cao, Wenjie Huang, Yichen Guo, Xijiu Jin

**Affiliations:** Department of Animal Science, College of Agriculture, Yanbian University, Yanji 133002, China; 18082170193@163.com (Y.L.); 18345803606@163.com (W.C.); 17767739890@163.com (W.H.); 13944675482@163.com (Y.G.)

**Keywords:** intestinal health, broilers, immunity, antioxidant

## Abstract

Maintaining intestinal health is fundamental to immune competence and disease resistance in broiler chickens; however, effective nutritional strategies that enhance gut barrier integrity and systemic health remain under investigation. This study evaluated the effects of a multi-strain probiotic composed of *Lactobacillus buchneri*, *Lactobacillus casei*, *Lactobacillus fermentum*, *Lactiplantibacillus plantarum*, and *Bacillus subtilis* on growth performance, meat quality, immune response, antioxidant capacity, and intestinal health in broilers. The research included 144 Ross 308 broilers aged one day, distributed randomly into two diet groups, each containing 6 replicates of 12 birds. During the 42 d trial, the control group (CON) was fed a basal diet composed of corn and soybean meal, with the compound probiotics (CP) diet additionally containing 0.3% compound microbial preparation. Growth performance, meat quality, serum immune and antioxidant indices, intestinal morphology, intestinal barrier-related genes and cecal microbiota were analyzed. Compared with the CON, CP supplementation significantly increased average daily gain, reduced feed conversion ratio, and improved eviscerated and breast muscle yields (*p* < 0.05). Meat quality was enhanced, as indicated by higher pH_45min_, increased redness (a*), and reduced shear force and drip loss in breast muscle (*p* < 0.05). In addition, CP broilers exhibited improved amino acid and fatty acid profiles in breast muscle (*p* < 0.05). Serum analysis revealed elevated immunoglobulin G and interleukin-10 levels, reduced pro-inflammatory cytokines, and enhanced antioxidant capacity in the CP group (*p* < 0.05). Probiotic supplementation significantly improved intestinal morphology and upregulated barrier-related genes such as mucin-1 (*MUC-1*), claudin-1(*CLDN-1*), zonula occludens-1 (*ZO-1*), and occludin (*OCLN*) (*p* < 0.05). Moreover, cecal microbiota analysis demonstrated increased abundance of Bacteroidota and enrichment of beneficial genera, including *Megamonas*, *Ruminococcus*, and *Prevotella* (*p* < 0.05). Overall, dietary supplementation with 0.3% compound probiotics effectively enhanced growth performance, immune function, antioxidant capacity, and intestinal health in broilers.

## Simple Summary

Maintaining good intestinal health is essential for disease resistance and overall health in broiler chickens. Probiotics are commonly used in poultry production, but the combined effects of specific multi-strain probiotics on growth, meat quality, immunity, and gut health are not fully understood. In this study, we evaluated a compound probiotic containing five beneficial microorganisms and its effects on broiler chickens during a 42-day feeding trial. Broilers receiving the probiotic supplement showed improved growth performance and feed efficiency, as well as better meat quality, including increased tenderness and water-holding capacity. Probiotic supplementation also enhanced immune responses and antioxidant capacity while reducing inflammatory markers in the blood. In addition, the probiotic improved intestinal structure, strengthened the gut barrier, and favorably altered the composition of intestinal microbiota. Overall, our findings indicate that dietary supplementation with this multi-strain probiotic can effectively support intestinal health, immune function, and overall performance in broiler chickens, suggesting its potential as a practical nutritional strategy to promote poultry health and productivity.

## 1. Introduction

Antibiotics, commonly referred to as antibiotic growth promoters (AGPs), have long been incorporated into animal feeds for their broad-spectrum antimicrobial properties. However, since their adoption, the effects of antibiotics have extended to all stages of food production and processing [1]. The broad application of antibiotics, in particular, has accelerated the emergence and proliferation of pathogens resistant to antimicrobials, which significantly endangers animal and public health [2]. Some experts have even described the problem of antibiotic resistance as an “apocalyptic threat” [3]. The European Union and the United States implemented the ban on AGPs and the Veterinary Feed Directive (VFD) regulation in 2006 and 2017, respectively, thereby prohibiting the use of antibiotics in feed for growth promotion purposes. In China, the production, sale, and use of antibiotic growth promoters in animal feed were officially discontinued from 1 July 2020 [4]. Therefore, identifying sustainable and safe feed additives to replace antibiotics is urgently needed to maintain productivity and product quality in animal production.

Broilers are widely reared in intensive production systems owing to their rapid growth, high carcass yield, and superior feed efficiency. Nevertheless, challenges such as high-density rearing and exposure to pathogens can directly impair broiler growth and contribute to the development of intestinal disorders. With the prohibition of antibiotic use, production costs have increased and disease control pressure has intensified, prompting researchers to shift their focus toward environmentally friendly, efficient, and residue-free microbial additives as potential alternatives [5].

In recent years, probiotics have gained increasing attention in livestock production. The positive impacts of microbial-based preparations are commonly explained through four key mechanisms: competitive exclusion, enhanced nutrient digestion and absorption, immunomodulation, and detoxification of harmful compounds [6]. Therefore, probiotic strains with complementary functional properties are often combined to achieve broader and more stable biological effects. In the present study, strains were selected based on previous reports regarding their roles in regulating the gut environment, improving nutrient utilization, and supporting intestinal health. Specifically, *Lactobacillus buchneri* can convert lactic acid into acetic acid, which helps maintain an acidic environment that suppresses the growth of undesirable microorganisms [7]; *Lactobacillus casei* has been associated with enhanced intestinal epithelial barrier integrity [8]; *Lactobacillus fermentum* and *Lactiplantibacillus plantarum* exhibit strong tolerance to gastrointestinal stress and can modulate the intestinal microenvironment through organic acid production, thereby promoting microbial balance and antimicrobial activity [9,10]; and *Bacillus subtilis* was included for its ability to form stress-resistant spores and secrete digestive enzymes [11]. In brief, *B. subtilis* may support oxygen consumption and digestive processes, whereas lactic acid bacteria facilitate intestinal colonization, environmental regulation, and pathogen suppression, collectively contributing to gut function and immune homeostasis and thereby improving feed digestion and nutrient utilization. Research has shown that *Bacillus subtilis*-based microbial preparations can perform comparably to antibiotic treatments in maintaining gut microbiota homeostasis, improving intestinal morphology, and increasing feed efficiency [12]. Additionally, strains such as *Lactobacillus*, *Bifidobacterium*, and *Enterococcus* are also commonly used in production [13]. Reuben et al. demonstrated that, compared with single-strain probiotics, multi-strain formulations more effectively improved growth performance while reducing serum cholesterol and glucose concentrations and increasing hemoglobin levels, along with increases in hemoglobin, white blood cells, and platelets, indicating better comprehensive effects of compound probiotics on immunomodulation and digestive improvement [14]. In line with this, Zhang et al. [15] observed that a combination of probiotics, including *Lactobacillus acidophilus*, *Bacillus subtilis*, and *Clostridium butyricum*, boosted humoral immunity and adjusted the cecal microbiota composition. Furthermore, compound probiotics have demonstrated potential in mitigating oxidative stress and supporting performance under environmental challenges such as heat stress [16].

Therefore, the present study aimed to systematically evaluate the efficacy of a compound probiotic as an antibiotic alternative in broiler production through an integrated analysis of growth performance, meat quality, serum biochemical parameters, intestinal morphology, intestinal barrier-related gene expression, and cecal microbiota composition, thereby providing a theoretical basis for microbiota-mediated regulation of host physiology. Notably, the supplementation level of 0.3% for the compound probiotic preparation was selected based on previous studies and our preliminary trial, with consideration of practical feasibility and cost effectiveness [17,18]. The preliminary results indicated that this inclusion level improved growth performance without affecting feed consumption.

## 2. Materials and Methods

### 2.1. Animal Ethics

This study adhered to animal experimental ethics standards and was approved by the Animal Ethics Committee of Yanbian University to protect the animals used in scientific research (Approval No. YD20240827010).

### 2.2. Probiotic Preparation

The probiotic preparation used in this study was produced by the Biofermentation Laboratory of Yanbian University. It consisted of *Lactobacillus buchneri*, *Lactobacillus casei*, *Lactobacillus fermentum*, *Lactiplantibacillus plantarum*, and *Bacillus subtilis* mixed at an equal ratio. The preparation procedure was as follows: corn flour was used as the main solid carrier and was thoroughly sterilized prior to inoculation with *L. buchneri*, *L. casei*, *L. fermentum*, *L. plantarum*, and *B. subtilis* at a ratio of 1:1:1:1:1. Solid-state fermentation was then carried out under anaerobic conditions at 37 °C for 48 h. After fermentation, the product was vacuum-dried at low temperature (35–40 °C) to rapidly remove moisture. The dried material was subsequently ground and sieved to obtain a powdered solid probiotic preparation. The viable cell count of the probiotic preparation used in this study was determined using the standard plate count method. Briefly, the sample was serially diluted tenfold under sterile conditions. Lactic acid bacteria were plated on De Man, Rogosa, and Sharpe (MRS) agar (Solarbio, Beijing, China), while *B. subtilis* was plated on nutrient agar (Solarbio, Beijing, China). The plates were incubated at 37 °C under anaerobic conditions for lactic acid bacteria and aerobic conditions for *B. subtilis*. After incubation, plates containing 30–300 colony-forming units (CFUs) were selected for enumeration, and the viable cell count was calculated based on the dilution factor and expressed as CFU/g. The viable cell concentration of the probiotic preparation was determined to be 3 × 10^8^ CFU/g. Moreover, the probiotic preparation was directly mixed into the daily diet, and the feed was prepared fresh each day and offered immediately; thus, probiotic survival during extended feed storage was not evaluated.

### 2.3. Experimental Animals and Design

A group of 144 one-day-old male Ross 308 broilers were randomly divided into two diet groups: one receiving a basic diet (CON) and the other receiving the same basic diet supplemented with 0.3% compound probiotics (CP). A total of 72 birds per treatment were distributed across six replicates (12 birds per replicate). Broilers were reared in a step-cage system for 42 days. Individual birds were identified using leg bands. Before commencing the trial, all cages and equipment were meticulously cleaned and disinfected. Temperature started at 33 °C and was lowered weekly (2–3 °C) until 24 °C. Uninterrupted lighting and consistent airflow were maintained during the study. The birds could freely access food and water, and they received vaccinations according to a regular schedule.

### 2.4. Diet Composition and Nutrition Level Analysis

The basal diet was formulated to meet the nutrient specifications for Ross 308 broilers, with NRC (1994) [19] consulted as a supplementary reference for ingredient nutrient composition. The experimental diet samples were analyzed according to the recommended national standard of China (GB/T): crude protein (GB/T 6432-2018, Kjeldahl method, N × 6.25) [20], calcium (GB/T 6436-2018, spectrophotometry after acid digestion) [21] and phosphorus (GB/T 6437-2018, spectrophotometric method) [22], and amino acids (GB/T 18246-2019, high-performance liquid chromatography) [23]. The ingredient composition and nutrient levels are shown in Table 1.

### 2.5. Sample Collection

On day 42, after 12 h of feed withdrawal, one bird per replicate was selected for sampling, giving six birds per treatment and 12 birds in total; birds were chosen based on having a body weight closest to the replicate mean. Whole blood was collected from the wing vein, allowed to clot at room temperature for 30 min, and then centrifuged (Micro 21R, Thermo Fisher Scientific Inc., Waltham, MA, USA) at 3000× *g* for 15 min at 4 °C to obtain serum. Serum was aliquoted into sterile cryovials and immediately stored at −80 °C for subsequent biochemical analyses. Birds were stunned by electrical shock and euthanized by exsanguination via the carotid artery. Following Madej et al. [24], the abdominal cavity was opened immediately to expose the gastrointestinal tract, and approximately 2 cm segments from the mid-duodenum, mid-jejunum, and mid-ileum were collected. The segments were gently flushed with pre-chilled sterile saline to remove luminal contents and fixed in 4% paraformaldehyde at room temperature for 24–48 h for histological processing and morphological evaluation. For gene expression analysis, a section of ileum was opened longitudinally, rinsed with pre-chilled saline, and the mucosa was gently scraped using a sterile coverslip. The mucosal samples were snap-frozen in liquid nitrogen, weighed, and stored at −80 °C for analysis of intestinal barrier-related gene expression. The ceca were then excised, and the entire cecal contents were collected into sterile centrifuge tubes, immediately frozen in liquid nitrogen, and stored at −80 °C for subsequent microbiota community analysis.

### 2.6. Measurement of Growth Performance

Body weight was recorded after fasting on days 1, 21, and 42. Average daily feed intake (ADFI) was calculated from pen-level daily consumption. Average daily gain (ADG) and feed conversion ratio (FCR) were determined for the starter (1–21 d), grower–finisher (22–42 d), and overall (1–42 d) phases, with FCR values adjusted for mortality by dividing total feed intake per pen by total body weight gain, calculated as the final live body weight of surviving birds plus the body weight of birds at the time of death minus the initial body weight.

### 2.7. Measurement of Carcass Performance

Prior to slaughter, live body weight was recorded. After slaughter and defeathering by wet plucking, the cuticle of the feet, toenails, and beak sheath were removed to obtain dressed weight. Subsequently, the reproductive organs, intestines, esophagus, trachea, crop, pancreas, spleen, gallbladder, gizzard contents, and cuticle were removed to determine semi-eviscerated weight. For eviscerated weight determination, the gizzard, proventriculus, liver, heart, lungs, abdominal fat, head, and feet were removed, with abdominal fat collected and weighed separately, following the procedure described by Xing et al. [25]. Breast and thigh muscles were dissected bilaterally from the carcass and individually weighed. Dressed, semi-eviscerated, eviscerated, breast muscle, thigh muscle, and abdominal fat yields were expressed relative to live weight. Specific methods referred to “Terms and Measurement Statistical Methods of Poultry Production Performance (NY/T 823-2020) [26].”

### 2.8. Measurement of Conventional Meat Quality Indicators

Breast muscle samples collected immediately after slaughter were used for the assessment of meat quality. Based on the method reported by Al-Owaimer et al. [27], the muscle’s pH was assessed 45 min post-mortem (pH_45min_) and again after 24 h at 4 °C (pH_24h_) utilizing a calibrated portable pH meter (ST3100, OHAUS Instruments, Changzhou, China), with the probe inserted about 40 mm into the muscle to maintain a consistent measurement depth. Following 24 h of chilling, breast muscle color was measured with a handheld colorimeter (CR-10, Konica Minolta Sensing, Shangahi, China). Measurements for lightness (L*), redness (a*), and yellowness (b*) were taken at three random spots on each sample and averaged. Samples were suspended in sealed bags at 4 °C for 24 h and re-weighed to calculate drip loss; cooking loss was determined after vacuum-sealing samples and heating in an 80 °C water bath to 70 °C internal temperature. After cooking, samples were cooled, surface-moisture blotted, and re-weighed to calculate cooking loss as percentage weight reduction. From each cooked sample, a strip measuring 3 × 1 × 1 cm was cut parallel to the orientation of the muscle fibres, and shear force was determined perpendicular to the fibre direction using a tenderness meter (C-LM3B, Tenovo International Co., Ltd., Beijing, China).

### 2.9. Analysis of Fatty Acids and Amino Acids

Meat samples stored on dry ice were submitted to the Jilin Academy of Agricultural Sciences for analysis. Fatty acid analysis was conducted via gas chromatography following the national standard GB 5009.168-2016 [28] “Determination of Fatty Acids in Meat and Meat Products.” Amino acid composition and content in chicken meat were determined with an automatic amino acid analyzer (A300, Dalian Elite Analytical Instruments Co., Ltd., Dalian, China) according to the national standard GB 5009.124-2016 [29] “Determination of Amino Acids in Foods.”

### 2.10. Measurement of Serum Biochemical Parameters

An automated clinical chemistry analyzer (BS-420, Shenzhen Mindray Bio-Medical Electronics Co., Ltd., Shenzhen, China) was used to measure serum biochemical indices such as total protein (TP), albumin (ALB), globulin (GLB), total cholesterol (TC), triglycerides (TGs), creatinine (CREA), urea (UREA), aspartate aminotransferase (AST), and alanine aminotransferase (ALT). Immune and antioxidant biomarkers were quantified using a microplate reader (DR-200BS, WuXi HiWell-Diatek Instruments Co., Ltd., Wuxi, China). Immune markers assessed included immunoglobulins A, G, and M (IgA, IgG, IgM) and cytokines such as tumor necrosis factor-α (TNF-α), interleukin-1β (IL-1β), interleukin-6 (IL-6), interleukin-8 (IL-8), and interleukin-10 (IL-10). Antioxidant status was determined by analyzing superoxide dismutase (SOD), glutathione peroxidase (GSH-Px), and catalase (CAT) activities, along with total antioxidant capacity (T-AOC) and malondialdehyde (MDA) levels.

### 2.11. Measurement of Small Intestinal Morphology

According to Liu et al. [30], following fixation, intestinal tissue samples were sequentially dehydrated through a graded ethanol series, cleared with xylene, embedded in paraffin, and sectioned. The resulting sections were stained with hematoxylin and eosin (H&E) and examined using a light microscopy imaging system (Carl Zeiss, Microsystems, Carl Zeiss AG, Oberkochen, Germany). Villus height and crypt depth were measured using Image-Pro Plus software (v6.0; Media Cybernetics, Rockville, MD, USA), and the villus height-to-crypt depth ratio (VH:CD) was subsequently calculated.

### 2.12. Real-Time Quantitative PCR

Following the manufacturer’s instructions, ileal mucosal RNA was extracted with TRIzol reagent (Invitrogen Life Technologies, Carlsbad, CA, USA). Using the RevertAid First Strand cDNA Synthesis Kit (Thermo Fisher Scientific, Waltham, MA, USA), complementary DNA was created through reverse transcription. cDNA was amplified by quantitative real-time PCR on a SLAN-96P system (Shanghai Hongshi Medical Technology Co., Ltd., Shanghai, China) using FastStart Universal SYBR Green Master (Rox) (Roche Diagnostics GmbH, Mannheim, Germany). The reaction setup, with a total volume of 25 μL, included 12.5 μL of 2× SYBR Green Master Mix, 1 μL of cDNA template, 1 μL each of the forward and reverse primers (Invitrogen Biotechnology Co., Ltd., Shanghai, China), and was filled to volume with nuclease-free water (HyPure™ Molecular Biology Grade Water; HyClone, Logan, UT, USA). Amplification included a 2 min denaturation at 95 °C, 40 cycles of 95 °C/15 s and 60 °C/30 s, and a melt-curve analysis (45–90 °C) to confirm specificity. Primers (Table 2) were synthesized by Beijing Huaying Biotechnology Research Institute. Using the 2^−ΔΔCt^ method with *β*-*actin* as the reference gene, expressions were calculated. Sterile, nuclease-free consumables (Axygen Biosciences, Union City, CA, USA) were used throughout.

### 2.13. Analysis of Cecal Microbiota

Total microbial DNA was extracted from cecal contents with the BeaverBeads^®^ Stool DNA Kit (Beaver Biomedical Engineering Co., Ltd., Suzhou, China) and assessed by 1.0% agarose gel electrophoresis (Sangon Biotech, Shanghai, China). Primers 338F (5′-ACTCCTACGGGAGGCAGCA-3′) and 806R (5′-GGACTACHVGGGTWTCTAAT-3′) were used to amplify the V3–V4 hypervariable region of the bacterial 16S rRNA gene. The resulting amplicons were purified using a MolPure^®^ Fast Gel Extraction Kit (Yeasen Biotechnology (Shanghai) Co., Ltd., Shanghai, China), and sequencing libraries were subsequently prepared. Following quality control, libraries meeting the required standards were subjected to sequencing on an Illumina NovaSeq 6000 platform (Illumina, San Diego, CA, USA). Raw reads were processed in Quantitative Insights Into Microbial Ecology 2 (QIIME2, v2020.6). To obtain amplicon sequence variants (ASVs), the Divisive Amplicon Denoising Algorithm 2 (DADA2) algorithm was applied for denoising, paired-end read merging, and chimera filtering. Chao1, Shannon, ACE and Simpson indices measured alpha diversity; beta diversity was analyzed via principal coordinates analysis (PCoA) with Binary Jaccard and Bray–Curtis distance matrices. Using the classify-consensus-blast method in QIIME2 against the SILVA database, taxa were assigned and their relative abundances reported as percentages.

### 2.14. Statistical Analysis

Data management and preliminary analysis were conducted in Microsoft Excel 2023, and SPSS (version 17.0; SPSS Inc., Chicago, IL, USA) was employed for statistical testing. Normality was assessed using the Shapiro–Wilk test, and homogeneity of variances was evaluated using Levene’s test. Differences between the two treatments were analyzed using an independent-sample *t*-test when assumptions were met. Data are presented as mean ± SD, with *p* < 0.05 considered statistically significant.

## 3. Results

### 3.1. Growth Performance

Table 3 shows that adding compound probiotics (CP) to the diet increased average daily gain (ADG) and decreased feed conversion ratio (FCR) compared to the control (CON) in the starter (1–21 days) and grower–finisher (22–42 days) phases (*p* < 0.05). Average daily feed intake (ADFI) did not differ between treatments in either period (*p* > 0.05). Over the 42-day period, birds on CP demonstrated a significantly increased final body weight (BW) and overall ADG (*p* < 0.05), paired with a significantly lower cumulative FCR (*p* < 0.05), yet overall ADFI remained the same between the groups (*p* > 0.05).

### 3.2. Carcass Performance

Table 4 outlines the carcass traits. The CP group broilers showed significantly higher eviscerated and breast muscle yields compared to the CON (*p* < 0.05). The CP group had numerically higher slaughter and thigh muscle yields; however, these increases were not significant (*p* > 0.05) and only represented a marginal trend (*p* < 0.1).

### 3.3. Meat Quality

#### 3.3.1. Conventional Meat Quality

As shown in Table 5, dietary supplementation with compound probiotics significantly increased breast muscle pH at 45 min postmortem (pH_45min_) and a* compared with the control group (*p* < 0.05). Compared with the CON, shear force and drip loss were significantly reduced in the CP group (*p* < 0.05).

#### 3.3.2. Amino Acids

In Table 6, the compound probiotic supplementation significantly altered the amino acid composition of breast muscle. The CP group showed higher glutamate and methionine but lower phenylalanine than the CON (*p* < 0.05).

#### 3.3.3. Fatty Acids

According to the data in Table 7, the incorporation of compound probiotic supplements brought about a notable shift in the fatty acid profile of the breast muscle tissue. The CP group demonstrated markedly elevated levels of palmitoleic, oleic, and linolenic acids in comparison to the control group (*p* < 0.05).

### 3.4. Serum Biochemical Parameters

According to Table 8, the CP group of birds had notably higher levels of TBA, IgG, IL-10, SOD, GSH-Px, CAT, and T-AOC compared to the CON (*p* < 0.05). In broilers receiving CP supplementation, there was a significant reduction in the levels of MDA, TG, AST, and the pro-inflammatory cytokines TNF-α, IL-6, and IL-8 (*p* < 0.05).

### 3.5. Small Intestinal Morphology

In Table 9, it is summarized that the villus height in the duodenum, jejunum, and ileum was significantly larger in the CP group than in the CON (*p* < 0.05). The VH:CD ratio was significantly elevated in all three intestinal segments of birds on the CP diet (*p* < 0.05), with no significant differences in crypt depth between the treatments (*p* > 0.05).

### 3.6. Expression of Intestinal Barrier Genes

**Figure 1:**
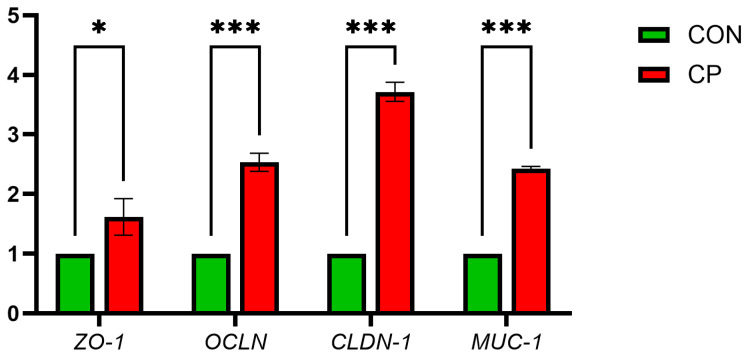
Effects of Dietary Supplementation with Compound Probiotics on the Expression of Intestinal Barrier Genes in Broilers. *ZO-1*, zonula occludens-1; *OCLN*, occludin; *CLDN-1*, claudin-1; *MUC-1*, mucin-1. The asterisk indicates statistical significance: * *p* < 0.05; *** *p* < 0.001; CON, control group; CP, compound probiotics.

Figure 1 illustrates that compared with CON, the CP group showed significant upregulation of ileal mucosal barrier genes *MUC-1*, *CLDN-1*, *ZO-1* and *OCLN* (*p* < 0.05).

### 3.7. Cecal Microbiota

#### 3.7.1. Quality Control Determination of Sequencing

**Figure 2:**
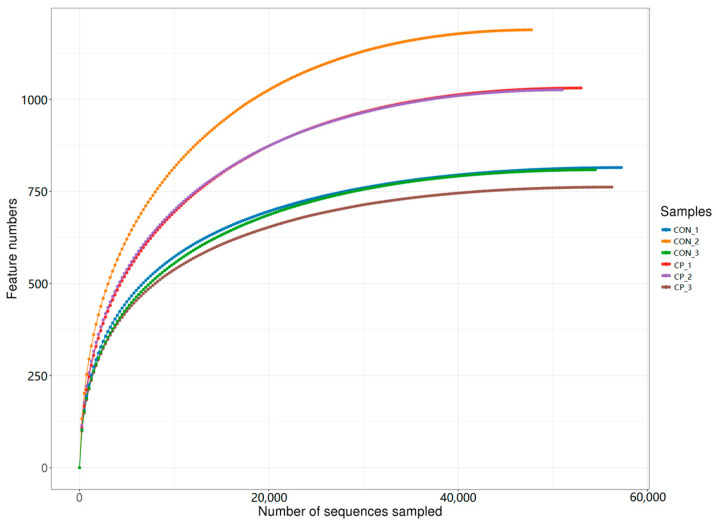
Rarefaction curves of the cecal microbiota samples. CON, control group; CP, compound probiotics. Lines represent individual samples from each treatment group.

After sequencing, a total of 479,642 paired-end reads were generated from the six cecal samples. Following quality filtering and read merging, 443,876 high-quality reads were retained, with at least 73,452 reads per sample and an average of 73,979 reads per sample. Rarefaction curves were generated by random subsampling and plotting sequencing depth against the number of observed features to assess whether sequencing effort adequately captured community richness. As shown in Figure 2, the rarefaction curves approached a plateau, indicating that the sequencing depth was sufficient to support subsequent diversity analyses in this dataset.

#### 3.7.2. Alpha Diversity and Beta Diversity Analyses

**Figure 3:**
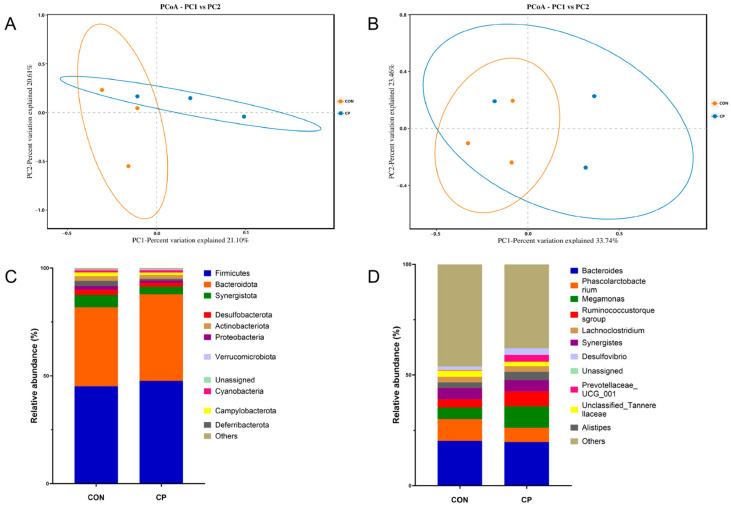
Principal Coordinates Analysis (PCoA) of the Cecal Microbiota in Broilers Based on Binary-Jaccard (**A**) and Bray–Curtis (**B**) Distance Matrices; Effects of Dietary Supplementation with Compound Probiotics on the Composition of the Cecal Microbiota in Broilers at the Phylum (**C**) and Genus (**D**) Levels; Orange circles represent the CON group, and blue circles represent the CP group; CON, control group; CP, compound probiotics.

Table 10 revealed no significant differences between CP group and CON in α-diversity metrics (Chao1, ACE, Shannon, and Simpson) (*p* > 0.05). By contrast, β-diversity analysis revealed distinct microbial community structures between treatments, as indicated by clear separation in principal coordinates analysis (PCoA) based on Binary-Jaccard (Figure 3A) and Bray–Curtis (Figure 3B) distance matrices. At the phylum level (Figure 3C), Firmicutes and Bacteroidota were predominant in the cecal microbiota. The CP group displayed significantly elevated Bacteroidota levels relative to their CON counterparts (*p* < 0.05), whereas Firmicutes demonstrated a modest uptick that did not quite reach statistical significance (*p* < 0.1). Significant enrichment of *Megamonas*, *Ruminococcus*, and *Prevotella* was observed at the genus level in broilers supplemented with CP (Figure 3D) (*p* < 0.05).

## 4. Discussion

### 4.1. Effects of Compound Probiotics on Growth Performance

Growth performance is closely related to the economic efficiency of animal production. Research on the application of microbial preparations as growth enhancers for broilers has been well documented. Probiotics are recognized to improve broiler performance mainly via modulation of the intestinal microbiota and regulation of host immune responses. Supporting this concept, Wang et al. [31] reported that dietary supplementation with *Lactobacillus plantarum* markedly enhanced body weight and feed conversion efficiency in broilers, while Yang et al. [32] found that inclusion of *Clostridium butyricum* significantly increased average daily gain (ADG). Consistent with these findings, the compound probiotic used in the present study significantly increased ADG and reduced feed conversion ratio (FCR) (*p* < 0.05), without affecting average daily feed intake (ADFI), indicating an improvement in feed utilization efficiency. It is worth noting that although probiotic supplementation significantly reduced the FCR in the present study, the FCR values remained higher than the commercial performance objectives reported in the Ross manual. This discrepancy is most likely attributable to differences in husbandry conditions and in the calculation definition, including the approach used for mortality adjustment. Importantly, all pens were managed under identical conditions, and the significantly lower FCR in the CP group than in the CON clearly indicates a positive effect of the compound probiotic on broiler growth performance and feed efficiency. However, positive growth responses to probiotic supplementation have not been consistently reported across studies. For example, Olnood et al. [33] observed no significant effects of lactic acid bacteria on broiler growth, which may be attributed to differences in broiler genotype, management practices, probiotic strains, or supplementation levels. The growth-promoting effects observed in the present study are likely associated with probiotic-mediated competitive exclusion of pathogenic microorganisms, together with improved intestinal digestion and nutrient absorption. From the perspective of cecal microbiota, the improved growth performance may also be linked to probiotic-induced shifts in microbial composition and metabolic potential. *Bacteroidota* are widely recognized for degrading complex polysaccharides and generating metabolites such as short-chain fatty acids (SCFAs), which can enhance energy harvest and nutrient utilization [34]. In the present study, the CP group showed a higher relative abundance of *Bacteroidota*, together with increases in genera involved in fiber and polysaccharide degradation, such as *Ruminococcus*, which may contribute to improved feed utilization. *Prevotella* is also closely related to carbohydrate metabolism and may support energy utilization through modulation of glucose-related pathways. In addition, the distinct separation in beta-diversity indicates an overall restructuring of the cecal microbial community, which could be associated with improved intestinal homeostasis and a lower inflammatory burden, thereby allowing more dietary energy to be allocated to growth. It should also be noted that only a single supplementation level (0.3%) of the compound probiotic was evaluated in this study. Although this dose was effective in improving the measured parameters, further studies investigating different inclusion levels are warranted to determine the optimal dosage for maximizing specific probiotic efficacy.

### 4.2. Effects of Compound Probiotics on Carcass Performance

In broiler production systems, carcass traits are essential metrics for evaluating efficiency [35]. Supplementation with compound probiotics significantly improved eviscerated and breast muscle yields (*p* < 0.05), reflecting elevated muscle deposition, especially in the breast. Similar to the observations for FCR, carcass performance parameters in the present study were slightly lower than the reference values reported in the Ross’s manual. This is consistent with the differences between the experimental conditions employed in this study and highly optimized commercial production systems, as discussed above. Nevertheless, under the same experimental conditions, dietary supplementation with the compound probiotic significantly increased eviscerated yield and breast muscle yield. These results indicate that, within the experimental context of the present study, the probiotic effectively promoted muscle deposition. Increasing evidence indicates that compound probiotics may improve growth performance and carcass characteristics by reshaping the cecal microbiota and its metabolic activity, thereby strengthening intestinal barrier function and supporting immune regulation [36]. In line with our findings, Chen et al. [37] observed that compound probiotics enhanced breast muscle and eviscerated yields in Lingshan broilers, confirming the potential of multi-strain probiotics to improve carcass traits.

### 4.3. Effects of Compound Probiotics on Meat Quality

In this trial, the compound probiotic supplementation demonstrated multiple beneficial impacts on broiler meat characteristics. Regarding conventional meat quality indicators, the pH_45min_ value was notably higher in the CP group compared to controls, suggesting that probiotic intervention slowed postmortem muscle acidification, thereby aiding in water retention and tenderness preservation. The significantly reduced drip loss further indicates better water-holding capacity in the CP group chicken meat. The significant decrease in shear force directly indicates improved meat tenderness, potentially related to changes in the activity of protein-degrading enzymes or the degree of collagen cross-linking in the muscle. Aksu et al. [38] found that supplementing broiler diets with *Saccharomyces cerevisiae* improved the pH value of chicken meat. Other studies have likewise demonstrated that probiotic supplementation can reduce shear force and improve water-holding capacity in poultry meat [39,40]. Moreover, the increased a* of breast muscle in the CP group aligns with previous reports indicating that probiotics can enhance meat color attributes favored by consumers [41,42]. In the present study, birds in the CP group showed significantly higher serum antioxidant enzyme activities, including SOD, GSH-Px, and CAT, as well as increased total antioxidant capacity, accompanied by a lower MDA level, indicating an enhanced systemic antioxidant defense. After slaughter, meat color is strongly influenced by myoglobin chemistry, and oxidative conversion of myoglobin to metmyoglobin leads to brown discoloration and a reduction in redness. The improved antioxidant status observed in the CP group may slow oxidative processes in muscle, thereby retarding myoglobin oxidation and helping maintain a higher proportion of oxygenated myoglobin. This could contribute to the elevated a* observed in the CP group. In contrast, some studies found probiotics had little to no impact on meat color, pH, or water-holding capacity [43,44]. Such discrepancies likely reflect differences in genetic background, management practices, slaughter conditions, and pre- and postmortem handling procedures [45].

Amino acids are critical precursors for flavor development and key determinants of nutritional quality in meat [46]; their composition and concentration profoundly influence eating characteristics [47,48]. Previous studies have shown that probiotics can alter the amino acid profile of broiler breast muscle. For instance, Tang et al. [49] found that dietary *Bacillus subtilis* supplementation significantly increased lysine, glutamate, methionine, and total essential amino acid content. Similarly, Liu et al. [41] reported that *Brevibacillus laterosporus* S62-9 enhanced multiple essential and flavor-associated amino acids. Consistent with these reports, the present study observed elevated concentrations of glutamate and methionine in breast muscle from the CP group. Glutamate is a major flavor-active amino acid that contributes to umami taste and overall palatability [50,51]. Methionine, as an essential amino acid, reflects improvements in overall protein metabolism. In addition, the reduced phenylalanine content observed in the CP group may be related to microbiota-mediated aromatic amino acid metabolism. Gut microorganisms can catabolize phenylalanine through aromatic amino acid metabolic pathways and convert it into downstream metabolites such as phenylacetic acid and other related aromatic compounds, which may reduce the availability of phenylalanine for deposition in host tissues [52]. Notably, Although certain amino acids showed apparent numerical differences between treatments, these differences were not statistically significant, likely due to relatively high inter-individual variability within groups.

The fatty acid composition is another essential factor governing meat quality and nutritional value [53]. The sensory characteristics and health effects are influenced by the relative amounts of saturated (SFAs), monounsaturated (MUFAs), and polyunsaturated fatty acids (PUFAs). Increased consumption of SFA is associated with higher cholesterol and low-density lipoprotein levels, which is why it is considered a risk factor for coronary artery disease [54]. In this study, the CP group showed markedly higher concentrations of palmitoleic, oleic, and linolenic acids. Oleic acid, a predominant MUFA, is associated with improved flavor and oxidative stability [55]. Poultry meat is an important dietary source of PUFAs, including α-linolenic and eicosapentaenoic acids [56]. α-Linolenic acid, an omega-3 fatty acid, exhibits anti-atherosclerotic and cardioprotective properties [57]. Its significant increase here suggests that the microbial preparation enhanced the nutritional profile of the meat. Furthermore, probiotic supplementation did not alter SFA content, indicating no adverse effect on meat quality. This pattern may reflect alterations in fatty acid remodeling rather than a reduction in saturated fatty acid synthesis. Because saturated fatty acids are both the primary products of endogenous lipogenesis and substrates for subsequent desaturation and elongation reactions, their absolute levels may remain relatively stable when synthesis and conversion occur concurrently. Previous studies have reported that probiotic intervention can increase stearoyl-CoA desaturase activity, and that this enzyme is closely associated with differences in unsaturated fatty acid, particularly PUFAs, content in broiler muscle [58]. Together, these results imply that the compound probiotic optimized intramuscular fat composition through modulation of lipid metabolic pathways.

### 4.4. Effects of Compound Probiotics on Serum Biochemical Parameters

Serum biochemical parameters provide valuable insight into broiler physiological status and overall health. Elevated TG levels are linked to metabolic syndrome and cardiovascular risk; therefore, reducing TGs may lower the incidence of metabolic disorders [59]. In this trial, TG concentrations were significantly lower in the CP group compared with the CON—a finding consistent with earlier work by Taslim et al., who reported that probiotic supplementation decreased serum TG in broilers [60]. Furthermore, bile acids, known regulators of lipid metabolism in poultry [61], were increased in the CP group, suggesting enhanced lipid metabolic activity. The elevated serum total bile acids in the CP group suggest that probiotic supplementation may have modulated bile acid metabolism and enterohepatic circulation through the gut–liver axis. In broilers, bile acids are key physiological emulsifiers required for lipid digestion and absorption, and increasing bile acid availability or turnover has been associated with improved fat digestibility and lipid utilization [62]. Beyond their digestive role, bile acids also act as metabolic signaling molecules, and bile acid signaling has been discussed in poultry in relation to regulation of lipid metabolism and inflammatory homeostasis via receptors such as FXR and TGR5 [63]. Liver function was assessed using the established biomarkers AST and ALT [64]. Lower AST levels correlate with enhanced liver function in clinical settings [65], and in our study, the CP group demonstrated markedly reduced enzyme activity (*p* < 0.05), suggesting improved hepatic well-being. This aligns with Bityutskyy et al. [66], who observed reduced ALT and AST in quails following probiotic administration. Humoral immunity was evaluated through serum immunoglobulins. Multiple studies indicate that probiotics can elevate immunoglobulin levels [32,67]. For instance, Perdigón et al. [68] showed that *Lactobacillus acidophilus* increased IgA-secreting cells, and Bai et al. [69] reported higher serum IgA and IgG in broilers supplemented with *Bacillus subtilis* fmbJ. In line with these reports, IgG was significantly increased in the CP group (*p* < 0.05), likely reflecting the immunomodulatory activity of the probiotic [70]. Cytokine profiles revealed a shift toward an anti-inflammatory state in the CP group, with higher IL-10 and lower TNF-α, IL-6, and IL-8 levels. IL-10 suppresses pro-inflammatory mediators [71], and the balance between anti- and pro-inflammatory cytokines is critical for effective pathogen defense [72,73]. These results, together with elevated IgG, suggest that the compound probiotic enhances immune function while mitigating inflammation. Oxidative stress increases MDA, damaging cell membranes and impairing health [74]. The damage is counteracted by antioxidant enzymes like SOD, GSH-Px, and CAT. Previous studies in quails [75] and broilers [76] have shown that probiotic supplementation boosts these enzymes and reduces MDA. Consistent with this, SOD, CAT, and GSH-Px activities were higher and MDA lower in the CP group. Compound probiotics may enhance antioxidant capacity partly through activation of the Nrf2 antioxidant response element signaling pathway. Upregulation of this pathway can increase the expression of key antioxidant enzymes such as SOD, GSH-Px, and CAT, thereby strengthening endogenous redox defense and contributing to the higher serum antioxidant enzyme activities and total antioxidant capacity [77], together with the lower MDA level, observed in the CP group. Moreover, T-AOC was also elevated, further confirming improved antioxidant status.

### 4.5. Effects on Intestinal Health

A key factor in poultry’s growth and immune system is the condition of their intestines. From a morphological perspective, increased villus height enhances digestive and absorptive capacity [78], while reduced crypt depth is generally indicative of improved epithelial turnover and maturation [79]. Consequently, VH:CD ratio is a direct indicator of small-intestinal functional efficiency, with higher values reflecting superior nutrient utilization in broilers. It should be noted that villus height is influenced by several factors, including bird age, diet composition, intestinal segment examined, and rearing conditions. In the present study, probiotic supplementation significantly increased villus height and the villus height-to-crypt depth ratio in the duodenum, jejunum, and ileum, with values exceeding those commonly reported for poultry. These findings suggest enhanced intestinal development and absorptive capacity under the specific experimental conditions employed. In contrast, crypt depth was not affected by probiotic supplementation. Crypt depth mainly reflects epithelial cell proliferation and tissue renewal, which may be less responsive to probiotics under relatively stable rearing conditions without marked intestinal stress. Therefore, probiotics may improve villus development and absorptive capacity without substantially altering crypt cell proliferation. Consistent with our findings, Chen et al. [80] also reported that *Bacillus subtilis* supplementation did not significantly affect crypt depth. Consistent with our results, Zhang et al. [81] reported that compound probiotics significantly improved villus architecture and villus height-to-crypt depth ratios, thereby promoting intestinal function. The intestinal epithelial barrier forms the primary defense against luminal pathogens, with tight junctions between enterocytes playing a critical role in maintaining barrier integrity [82]. Proteins such as *CLDN-1*, *OCLN*, and *ZO-1* are vital for maintaining barrier function, and their increased expression bolsters the integrity of the intestinal epithelium [83]. In this trial, expression of *CLDN-1*, *OCLN*, and *ZO-1* was significantly increased in the CP group, indicating reinforcement of the intestinal barrier. Furthermore, we observed a pronounced upregulation of *MUC-1*, a gene involved in mucin secretion, which suggests enhanced mucosal defense against pathogens [84]. Collectively, these findings align with Khan et al. [85], indicating that probiotics strengthen intestinal barrier function by modulating tight-junction protein expression.

The gastrointestinal microbiota performs essential functions in maintaining intestinal homeostasis, regulating immunity and metabolism, and is essential for ensuring the overall health of broilers [86]. Principal Coordinates Analysis (PCoA) revealed distinct separation between microbial communities from the CP group and CON, indicating significant differences in beta diversity between treatments. In both groups, *Firmicutes* and *Bacteroidota* were identified as the predominant phyla in the cecal microbiota. The relative abundance of *Bacteroidota* was markedly higher in broilers receiving CP treatment, whereas *Firmicutes* showed a tendency to increase. *Firmicutes* are closely implicated in systemic lipid metabolism, and the significant decrease in serum triglycerides noted in this study may be associated with lipid metabolic processes regulated by this phylum [87]. Literature reports indicate that elevated *Bacteroidota* abundance correlates with increased BW and improved FCR in broilers [88], which corresponds with our experimental findings. At the genus level, the CP group exhibited significantly higher abundances of *Megamonas*, *Ruminococcus*, and *Prevotella*. Research indicates that *Megamonas* abundance is positively correlated with PYGL gene expression., which participates in glycan metabolism in the cecum [89], suggesting that *Megamonas* may be related to energy metabolism in the host. *Ruminococcus* can increase the degradation of polysaccharides and fiber, thereby improving feed utilization, and generate short-chain fatty acids that support intestinal health [90]. *Prevotella* is closely associated with glucose metabolism [86]. Increased alpha diversity is generally considered beneficial for gut health, but not all microorganisms are beneficial, so higher diversity is not always better. Conversely, excessively high diversity might intensify microbial competition and disrupt community stability [91]. In this study, while alpha diversity remained unchanged, the enrichment of specific beneficial bacteria still conferred positive health effects on the broilers.

### 4.6. Limitations

This study evaluated a five-strain compound probiotic; thus, the observed effects represent the overall response to the blend and cannot be attributed to individual strains. Only one inclusion level was tested, and the preliminary dose screening was exploratory rather than a replicated dose–response trial, limiting conclusions on the optimal dosage. In addition, carcass traits, serum indices, intestinal morphology, gene expression, and microbiota analyses were conducted on 12 birds sampled at the end of the trial, with six birds per treatment, which may reduce statistical power and may not fully reflect within group variation. Future work should include replicated dose–response studies, assess individual strains and their interactions, monitor probiotic viability during feed storage, and increase sampling size to confirm these findings.

## 5. Conclusions

In summary, supplementing the diet with 0.3% compound probiotics significantly enhanced production performance, carcass characteristics, meat quality, serum biochemical profile, immune and antioxidant function, and intestinal health in broilers, while also modulating the cecal microbiota composition. This research provides theoretical foundations and empirical evidence supporting the application of compound probiotics as a viable, eco-friendly feed additive to replace antibiotics in broiler production systems.

## Figures and Tables

**Table 1:** Composition and Nutrient Levels of the Basal Diets.

Item	1–21 Day	22–42 Day
Ingredient composition (%) Corn	55.19	56.26
Soybean meal	31.29	28.44
Fish meal	2.00	2.00
Rapeseed meal	3.00	4.50
Soybean oil	3.00	4.00
Limestone	1.20	1.20
Dicalcium phosphate	1.50	1.30
Sodium chloride	0.30	0.30
Premix ^1^	2.00	2.00
Total	100	100
Nutrient levels ^2^		
Metabolizable energy (MJ/kg)	12.67	12.99
Crude protein (%)	19.7	19.03
Calcium (%)	0.93	0.87
Total phosphorus (%)	0.6	0.58
Available phosphorus (%)	0.28	0.26
Lysine (%)	1.08	1.03
Methionine (%)	0.34	0.32
Methionine + Cystine (%)	0.69	0.65

^1^ premix (per kg diet): The premix provided per kg of diet: Vitamin A 9000 IU, Vitamin D 500 IU, Vitamin E 35 IU, Vitamin K3 2.2 mg, Vitamin B1 2.00 mg, Vitamin B2 8.00 mg, Vitamin B6 3.50 mg, Vitamin B12 10.00 μg, Niacin 35.00 mg, Calcium pantothenate 10.00 mg, Folic acid 1 mg, Biotin 0.5 mg, Choline 1000 mg, Fe 80.00 mg, Cu 8.00 mg, Mn 100.00 mg, Zn 80.00 mg, I 0.70 mg, Se 0.30 mg. ^2^ Nutrient Level: The metabolizable energy and available phosphorus content were calculated values, while all other nutritional parameters were obtained through laboratory analysis.

**Table 2:** Primer Sequences and Parameters.

GeneBank	Primer Name	Primer Sequence (5′–3′)	Product Length (bp)
XM_040680628.2	*ZO-1*	TGTCAGCGCCTTTGTTCCTT GCCCCTATTGTCCGCAGG	87
NM_205128.1	*OCLN*	ATGCACCCACTGAGTGTTGG TGTGATGCTGTTGCAGACCT	125
NM_001013611.2	*CLDN-1*	AACGGCTAGCAAACTCCCAA CAATGCAGCAGCCCTAGAGA	106
XM_015279046.4	*MUC-1*	TTGTGGCTATGGGGATGCACT CCAGGCTGCCCCTCTTGTAT	167
NM_205518.2	*ACTIN*	CGGACTGTTACCAACACCCA TCCTGAGTCAAGCGCCAAAA	115

**Table 3:** Differences in growth performance of broilers between the CON and CP groups.

Items	CON	CP Group	*p*-Value
BW (g)			
1 d	45.6 ± 4.3	45.6 ± 3.6	0.97
21 d	732.1 ± 51.4	780.2 ± 87.7	0.082
42 d	2260.6 ± 180.5 ^b^	2418.0 ± 175.6 ^a^	0.03
1–21 d			
ADG	32.7 ± 2.0 ^b^	35.1 ± 0.9 ^a^	0.024
ADFI	49.1 ± 2.8	48.7 ± 2.4	0.79
FCR	1.503 ± 0.081 ^a^	1.389 ± 0.068 ^b^	0.033
22–42 d			
ADG	72.8 ± 2.5 ^b^	77.0 ± 1.9 ^a^	0.016
ADFI	137.9 ± 6.3	141.4 ± 1.2	0.221
FCR	1.895 ± 0.090 ^a^	1.813 ± 0.019 ^b^	0.045
1–42 d			
ADG	52.7 ± 1.09 ^b^	56.5 ± 0.83 ^a^	0.002
ADFI	93.5 ± 2.84	95.0 ± 0.57	0.229
FCR	1.774 ± 0.048 ^a^	1.683 ± 0.010 ^b^	0.002
Mortality (%)			
1–42 d	4.2	1.4	/

Values with different superscript letters (a, b) differ significantly (*p* < 0.05). CON, control group; CP, compound probiotics; BW, body weight; ADG, average daily gain; ADFI, average daily feed intake; FCR, feed conversion ratio; *n* = 6.

**Table 4:** Differences in Carcass Performance of broilers between the CON and CP groups (%).

Items	CON	CP Group	*p*-Value
Slaughter yield	89.99 ± 1.53	92.32 ± 0.82	0.081
Semi-eviscerated yield	80.42 ± 0.58	81.63 ± 1.34	0.224
Eviscerated yield	68.53 ± 1.13 ^b^	71.58 ± 0.73 ^a^	0.017
Breast muscle yield	19.94 ± 0.46 ^b^	22.51 ± 0.76 ^a^	0.007
Thigh muscle yield	18.78 ± 0.90	20.14 ± 0.45	0.079
Abdominal fat yield	3.91 ± 0.22	4.17 ± 0.04	0.115

Values with different superscript letters (a, b) differ significantly (*p* < 0.05). CON, control group; CP, compound probiotics; *n* = 6.

**Table 5:** Differences in conventional meat quality indicators of broilers between the CON and CP groups.

Items	CON	CP Group	*p*-Value
pH_45min_	6.57 ± 0.03 ^b^	6.72 ± 0.02 ^a^	0.002
pH_24h_	5.73 ± 0.06	5.84 ± 0.05	0.070
L*	51.00 ± 1.00	52.00 ± 1.00	0.288
a*	9.67 ± 0.58 ^b^	11.33 ± 0.58 ^a^	0.024
b*	11.00 ± 1.00	10.00 ± 1.00	0.288
Shear force (N)	35.43 ± 0.56 ^a^	26.19 ± 1.54 ^b^	0.001
Drip loss (%)	3.15 ± 0.16 ^a^	2.59 ± 0.14 ^b^	0.011
Cooking loss (%)	25.55 ± 3.66	20.48 ± 2.68	0.125
Water-holding capacity (%)	20.60 ± 1.86	20.57 ± 3.61	0.994

Values with different superscript letters (a, b) differ significantly (*p* < 0.05). L*, Lightness; a*, Redness; b*, Yellowness; CON, control group; CP, compound probiotics; *n* = 6.

**Table 6:** Differences in breast muscle amino acid content of broilers between the CON and CP groups (g/100 g).

Items	CON	CP Group	*p*-Value
Aspartic acid	1.92 ± 0.072	1.98 ± 0.081	0.471
Threonine	0.97 ± 0.023	0.99 ± 0.041	0.336
Serine	0.87 ± 0.020	0.9 ± 0.0350	0.158
Glutamic acid	3.24 ± 0.025 ^b^	3.37 ± 0.056 ^a^	0.024
Proline	0.68 ± 0.017	0.68 ± 0.040	0.806
Glycine	0.93 ± 0.006	0.97 ± 0.030	0.111
Alanine	1.22 ± 0.041	1.28 ± 0.050	0.167
Valine	0.93 ± 0.040	0.91 ± 0.040	0.573
Methionine	0.22 ± 0.101 ^b^	0.47 ± 0.015 ^a^	0.014
Isoleucine	0.90 ± 0.036	0.87 ± 0.030	0.501
Leucine	1.63 ± 0.032	1.69 ± 0.055	0.246
Tyrosine	0.72 ± 0.015	0.74 ± 0.017	0.155
Phenylalanine	1.14 ± 0.107 ^a^	0.90 ± 0.092 ^b^	0.043
Histidine	0.76 ± 0.159	0.76 ± 0.114	0.978
Lysine	1.8 ± 0.032	1.81 ± 0.097	0.832
Arginine	1.36 ± 0.032	0.91 ± 0.040	0.770
Total amino acids	19.37 ± 0.404	19.67 ± 0.665	0.541

Values with different superscript letters (a, b) differ significantly (*p* < 0.05). CON, control group; CP, compound probiotics; *n* = 6.

**Table 7:** Differences in breast muscle fatty acid content of broilers between the CON and CP groups (mg/g).

Items	CON	CP Group	*p*-Value
Methyl palmitoleate	0.0497 ± 0.01097 ^b^	0.1083 ± 0.01457 ^a^	0.005
Methyl heptadecanoate	0.0029 ± 0.00181	0.0023 ± 0.00162	0.713
Methyl stearate	0.0643 ± 0.01779	0.0747 ± 0.01845	0.875
Methyl elaidate	0.1303 ± 0.06757	0.0957 ± 0.03443	0.680
Methyl oleate	0.1047 ± 0.00451 ^b^	0.1787 ± 0.01704 ^a^	0.002
Methyl linoleate	0.0116 ± 0.00430	0.0147 ± 0.00257	0.354
Methyl linolenate	0.0020 ± 0.00051 ^b^	0.0038 ± 0.00051 ^a^	0.013

Values with different superscript letters (a, b) differ significantly (*p* < 0.05). CON, control group; CP, compound probiotics; *n* = 6.

**Table 8:** Differences in serum biochemical parameters of broilers between the CON and CP groups.

Items	CON	CP Group	*p*-Value
TP (g/L)	27.47 ± 2.86	25.42 ± 2.14	0.128
ALB (g/L)	13.71 ± 0.48	13.08 ± 0.99	0.129
GLB (g/L)	13.76 ± 2.67	12.34 ± 2.02	0.252
A/G	1.03 ± 0.22	1.08 ± 0.17	0.619
TC (mmol/L)	3.69 ± 0.37	3.48 ± 0.23	0.196
TG (mmol/L)	0.39 ± 0.06 ^a^	0.24 ± 0.03 ^b^	<0.001
AST (U/L)	364.43 ± 62.82 ^a^	293.26 ± 41.26 ^b^	0.018
ALT (U/L)	2.63 ± 0.88	2.02 ± 0.87	0.182
TBA (μmol/L)	2.88 ± 1.27 ^b^	5.72 ± 1.91 ^a^	0.004
IgA (g/L)	2.42 ± 0.68	2.31 ± 1.12	0.82
IgG (g/L)	3.52 ± 0.64 ^b^	4.74 ± 1.29 ^a^	0.031
IgM (g/L)	1.58 ± 0.59	1.79 ± 0.96	0.614
TNF-α (pg/mL)	65.79 ± 6.25 ^a^	43.57 ± 11.00 ^b^	<0.001
IL-1β (pg/mL)	24.40 ± 3.84	21.10 ± 2.36	0.057
IL-6 (pg/mL)	151.49 ± 17.73 ^a^	100.08 ± 18.19 ^b^	<0.001
IL-8 (pg/mL)	84.03 ± 5.30 ^a^	76.73 ± 4.94 ^b^	0.013
IL-10 (pg/mL)	45.39 ± 7.34 ^b^	55.81 ± 6.85 ^a^	0.011
MDA (nmol/mL)	2.84 ± 0.29 ^a^	2.47 ± 0.25 ^b^	0.015
SOD (U/mL)	75.86 ± 5.72 ^b^	87.73 ± 5.36 ^a^	0.001
GSH-PX (U/mL)	152.55 ± 19.43 ^b^	169.38 ± 9.09 ^a^	0.044
CAT (U/mL)	42.82 ± 3.53 ^b^	48.92 ± 6.94 ^a^	0.044
T-AOC (U/mL)	9.94 ± 0.67 ^b^	10.72 ± 0.71 ^a^	0.041

Values with different superscript letters (a, b) differ significantly (*p* < 0.05). CON, control group; CP, compound probiotics; TP, total protein; ALB, albumin; GLB, globulin; ALT, alanine aminotransferase; AST, aspartate aminotransferase; TGs, triglycerides; TBA, total bile acids; IgA, immunoglobulin A; IgG, immunoglobulin G; IgM, immunoglobulin M; TNF-α, tumor necrosis factor-α; IL, interleukin; MDA, Malondialdehyde; SOD, superoxide dismutase; GSH-PX, glutathione peroxidase; CAT, Catalase; T-AOC, total antioxidant capacity; *n* = 6.

**Table 9:** Differences in small intestinal morphology of broilers between the CON and CP groups (μm).

Items	CON	CP Group	*p*-Value
Duodenum			
Villus height	1600.13 ± 23.06 ^b^	1788.79 ± 10.47 ^a^	0.002
Crypt depth	161.02 ± 3.64	168.76 ± 2.31	0.147
VH:CD	9.94 ± 0.09 ^b^	10.60 ± 0.11 ^a^	0.009
Jejunum			
Villus height	1222.01 ± 13.36 ^b^	1404.13 ± 39.97 ^a^	0.012
Crypt depth	141.71 ± 2.82	134.33 ± 1.70	0.273
VH:CD	8.63 ± 0.14 ^b^	10.45 ± 0.24 ^a^	0.049
Ileum			
Villus height	1060.61 ± 24.39 ^b^	1165.47 ± 16.51 ^a^	0.013
Crypt depth	121.07 ± 3.65	124.98 ± 1.62	0.145
VH:CD	8.60 ± 0.07 ^b^	9.33 ± 0.22 ^a^	0.033

Values with different superscript letters (a, b) differ significantly (*p* < 0.05). CON, control group; CP, compound probiotics; VH:CD, villus height-to-crypt depth ratio; *n* = 6.

**Table 10:** Differences in alpha diversity of broilers between the CON and CP group.

Items	CON	CP Group	*p*-Value
ACE	939.28 ± 218.36	940.81 ± 154.07	0.993
Chao1	937.71 ± 217.70	939.69 ± 153.89	0.99
Simpson	0.97 ± 0.015	0.98 ± 0.005	0.742
Shannon	7.08 ± 0.64	6.96 ± 0.29	0.787

CON, control group; CP, compound probiotics; *n* = 3.

## Data Availability

The data that support the findings of this study are available on request: from the corresponding author. The data are not publicly available due to privacy or ethical.
